# Novel clinical biomarkers in blood and pleural effusion for diagnosing patients with tuberculosis distinguishing from malignant tumor

**DOI:** 10.1097/MD.0000000000031027

**Published:** 2022-10-14

**Authors:** Jian Wang, Zhe-Xiang Feng, Tao Ren, Wei-Yu Meng, Imran Khan, Xing-Xing Fan, Hu-Dan Pan, Liang Liu, Yi-Jun Tang, Xiao-Jun Yao, Run-Ze Li, Mei-Fang Wang, Elaine Lai-Han Leung

**Affiliations:** a Dr. Neher’s Biophysics Laboratory for Innovative Drug Discovery/State Key Laboratory of Quality Research in Chinese Medicine/Macau Institute for Applied Research in Medicine and Health, Macau University of Science and Technology, Macau (SAR), China; b Department of Respiratory and Critical Care Medicine, Taihe Hospital, Hubei University of Medicine, Hubei, China; c State Key Laboratory of Dampness Syndrome of Chinese Medicine, The Second Affiliated Hospital of Guangzhou University of Chinese Medicine (Guangdong Provincial Hospital of Chinese Medicine), Guangzhou, Guangdong, China; d Cancer Center, Faculty of Health Science, University of Macau, Macau (SAR), China; e MOE Frontiers Science Center for Precision Oncology, University of Macau, Macau (SAR), China.

**Keywords:** biomarker, cancerous, diagnosis, pleural effusion, tuberculous

## Abstract

Pleural effusion (PE) is a common manifestation of tuberculosis (TB) and malignant tumors but tuberculous PE (TPE) is difficult to distinguish from malignant PE (MPE), especially by noninvasive detection indicators. This study aimed to find effective detection indices in blood and PE for differentiating TB from a malignant tumor. A total of 815 patients who were diagnosed with TB or cancer in Hubei Shiyan Taihe Hospital from 2014 to 2017 were collected. Amongst them, 717 were found to have PE by thoracoscopy. Clinical characteristics, patients’ blood parameters and PE indicator information were summarized for analysis. Patients with MPE had higher percentages to be bloody and negative of Rivalta test in PE than those with TPE. For clinical indicators, comparison of the specific parameters in blood showed that 18 indicators were higher in the TPE group than in the MPE group. By contrast, 12 indicators were higher in the MPE group than in the TPE group (*P* < .01). In addition, in PE tests, 3 parameters were higher in the TPE group, whereas other 4 parameters were higher in the MPE group (*P* < .01). Then, for clinical diagnosing practice, ROC analysis and principal component analysis were applied. The top 6 relevant indicators with area under curve over 0.70 were screened out as follows: hydrothorax adenosine dehydrogenase (pADA, 0.90), hydrothorax high-sensitivity C reactive protein (0.79), percentage of blood monocyte (sMONp, 0.75), blood high-sensitivity C reactive protein (sHsCRP, 0.73), erythrocyte sedimentation rate (0.71) and blood D-dimer (0.70). Moreover, logistic regression model revealed that a specific combination of 3 biomarkers, namely, pADA, sMONp and sHsCRP, could enhance the distinguishment of TB from malignant tumor with PE (area under curve = 0.944, 95% confidence interval = 0.925–0.964). The diagnostic function of the top single marker pADA in patients from different groups was analyzed and it was found to maintain high specificity and sensitivity. The 6 indicators, namely, pADA, hydrothorax high-sensitivity C reactive protein, sMONp, sHsCRP, sESR and blood D-dimer, showed significant diagnostic value for clinicians. Further, the combination of pADA, sMONp and sHsCRP has high accuracy for differential diagnosis for the first time. Most interestingly, the single marker pADA maintained high specificity and sensitivity in patients with different statuses and thus has great value for rapid and accurate diagnosis of suspected cases.

## 1. Introduction

Pleural effusion (PE) is mainly seen in various types of inflammation, tuberculosis (TB), and malignant tumors.^[1–3]^ The onset of tuberculous PE (TPE) is more insidious,^[4]^ with slow course and lack of specificity.^[5]^ Malignant PE (MPE) is also common, with approximately 20% being the first symptom and 30% to 40% occurring in the course of the disease, indicating poor prognosis and short survival.^[6]^ Patients with MPE had worse prognosis than those without MPE (median survival of 7.49 vs 12.65 months, *P* < .001).^[6]^ Early diagnosis and treatment could lead to enhanced prognosis.

In the current clinical practice, isolation of mycobacterium TB in the pleural fluid is difficult and could be negative in the acute setting.^[7]^ However, the main obstacle in diagnosing malignant effusions is the presence of false-negative cytological results in approximately 40% of cases.^[8]^ More invasive procedures (such as pleural biopsy) to identify caseating granuloma from the parietal pleura may be required. Thoracoscopic surgery is decisive for TPE and MPE^[9]^ but it is not widely used because of its invasive property. Consequently, the development of noninvasive methods is important to differentially diagnose these 2 diseases.

Some noninvasive studies identified patients with TPE from those with malignant tumor.^[10–12]^ For instance, serum total protein, albumin and globulin were significantly higher in the TB group than in the lung cancer (CA) group, whilst serum lactate dehydrogenase (sLDH) was higher in the lung CA group than in the TB group (*P *< .01).^[13]^ Some researchers found that the serum D-dimer level of patients with TPE was higher than those of patients with MPE.^[14]^ In addition, lymphocytes and macrophages were the predominant nucleated cell in MPE and TPE was characterized by a large percentage of leukocytes and lymphocytes (*P* < .01).^[15]^ However, these results all meet the problems of low sensitivity and low specificity. In the present study, clinical data of 717 patients with TPE or MPE were investigated to analyze their clinical characteristics, hydrothorax parameters and blood parameters. This study has a large sample size. The significant indicators in the differential diagnosis were preliminarily expounded, which is beneficial to distinguish PE early and improve the accuracy of diagnosis.

## 2. Methods

### 2.1. Data collection

This research was a retrospective study. A total of 815 patients diagnosed with TB or CA in Hubei Shiyan Taihe Hospital between 2014 and 2017 were recruited in this study. All patients agreed to participate in the study and signed informed consent forms. They underwent thoracoscopic examination, and the results showed that 717 patients had PE. The age and gender characteristics of patients were shown in Table 1.

**Table 1:** Age and gender characteristics of the study population.

Features	Clinical diagnosis			F/X^2^
	Tuberculosis	Cancer	Total	*P*-value
Total	641	174	815	
With PE	570	147	717	
Without PE	71	27	98	
Gender				
Male	453	92	545	19.57
Female	188	82	270	.0001
Age				
<25	149	1	150	
25 to 45	245	15	260	162.18
45 to 65	191	102	293	.0001
>65	56	56	112	

PE = pleural effusion.

The inclusion criteria for TPE were as follows^[16]^: pathological examination revealed TB foci; positive for acid-fast staining or positive for the culture of mycobacterium TB and significant absorption of PE in anti-TB treatment. At least 1 of the above criteria should be met. The inclusion criteria for cancerous PE were as follows^[17]^: imaging examination showed thoracic mass shadow; PE was exudative; negative for acid-fast staining or negative for TB bacillus culture and the histological or cytological examination confirmed malignant tumor. All the above criteria must be met.

### 2.2. PE and blood statistical analysis

All data below were collected from Hubei Shiyan Taihe Hospital.

The PE analysis indicators included hydrothorax adenosine dehydrogenase (pADA), hydrothorax amylase, hydrothorax cell, hydrothorax glucose, hydrothorax high-sensitivity C reactive protein (pHsCRP), hydrothorax monocyte (pMON), hydrothorax nucleated cell, hydrothorax total cholesterol and hydrothorax total protein.

The blood analysis indicators included blood albumin, blood alkaline phosphatase, alanine aminotransferase, activated partial prothrombin time, aspartate aminotransferase, blood A/G (sA/G), percentage of blood basophil cell, serum calcium, blood creatine kinase, blood creatine kinase isoenzyme, blood chlorine, serum creatinine, blood D-dimer (sD-dimer), percentage of eosinophil, erythrocyte sedimentation rate, blood fibrin, fibrinogen degradation product, globin, percentage of blood granulocyte, blood bicarbonate, hemoglobin, blood high-sensitivity C reactive protein (sHsCRP), blood internationalization standardized ratio, serum kalium, sLDH, lymphocyte percentage, serum magnesium, percentage of blood monocyte (sMONp), blood natrium, serum phosphate, blood prealbumin, platelet, prothrombin time, prothrombin activity, red blood cell, prothrombin time ratio, total bilirubin, total bile acid, blood total protein, blood thrombin time, blood urea, white blood cell (sWBC), blood *α* hydroxybutyrate dehydrogenase and blood *γ* glutamyl transpeptidase.

Numerical analysis results showed that the data obeyed the normal distribution. SPSS 18.0 software was used for statistical analysis of count data by chi-square test and measurement data by independent *t* test. Comparison was analyzed using analysis of variance between groups. The ROC curve was used to determine the best threshold (cut off) and the area under curve (AUC) after series-parallel experiment. *P *< .05 was considered statistically significant.

## 3. Results

### 3.1. Demographic and clinical characteristics of study population

A total of 717 patients with TPE or MPE were recruited in this study. These patients were divided into 2 groups: MPE (147, 20.4%) and TPE (570, 79.6%). As shown in Table 2, multiple clinical status of patients and physical characteristics of the patient’s PE was analyzed to distinguish TPE from MPE. Amongst patients with hydrothorax, those with TB had a higher possibility to experience fever. Besides, patients with malignant tumor (40.0%) were more likely to have bloody PE than those with TB. The PE of patients with CA (67.3%) had a higher percentage to be turbid. Amongst patients with hydrothorax, those with TB (91.6%) had a higher percentage to be positive in Rivalta test (Table 2).

**Table 2:** Demographic and clinical characteristics of the study population.

Features	Clinical diagnosis		F/X^2^	
	Tuberculosis	Cancer	Total	*P*-value
**Hydrothorax**	641	174	815	
Yes	570 (88.9%)	147 (84.5%)	717	2.551
No	71 (11.1%)	27 (15.5%)	98	.11
**Fever**	570	147	717	
Yes	346 (60.7%)	18 (12.2%)	364	109.79
No	224 (39.3%)	129 (87.8%)	353	.0001
**Total Color of pleural effusion**	560	145	705 (12 missed)	
Yellow	471 (84.1%)	79 (54.5%)	550	73.21
Pink	30 (5.4%)	8 (5.5%)	38	.0001
Red	59 (10.5%)	58 (40.0%)	117	
**Total transparency of pleural effusion**	569	147	716 (1 missed)	
Turbid	270 (47.5%)	99 (67.3%)	550	18.52
Light turbid	182 (32.0%)	29 (19.7%)	38	.0001
Transparent	117 (20.6%)	19 (12.9%)	117	
**Rivalta test**	570	146	716(1 missed)	
Negative	48 (8.4%)	25 (17.1%)	73	9.61
Positive	522 (91.6%)	121 (82.9%)	643	.002

### 3.2. Discriminative indicators in clinical practice to identify TPE and MPE

**Figure 1.**
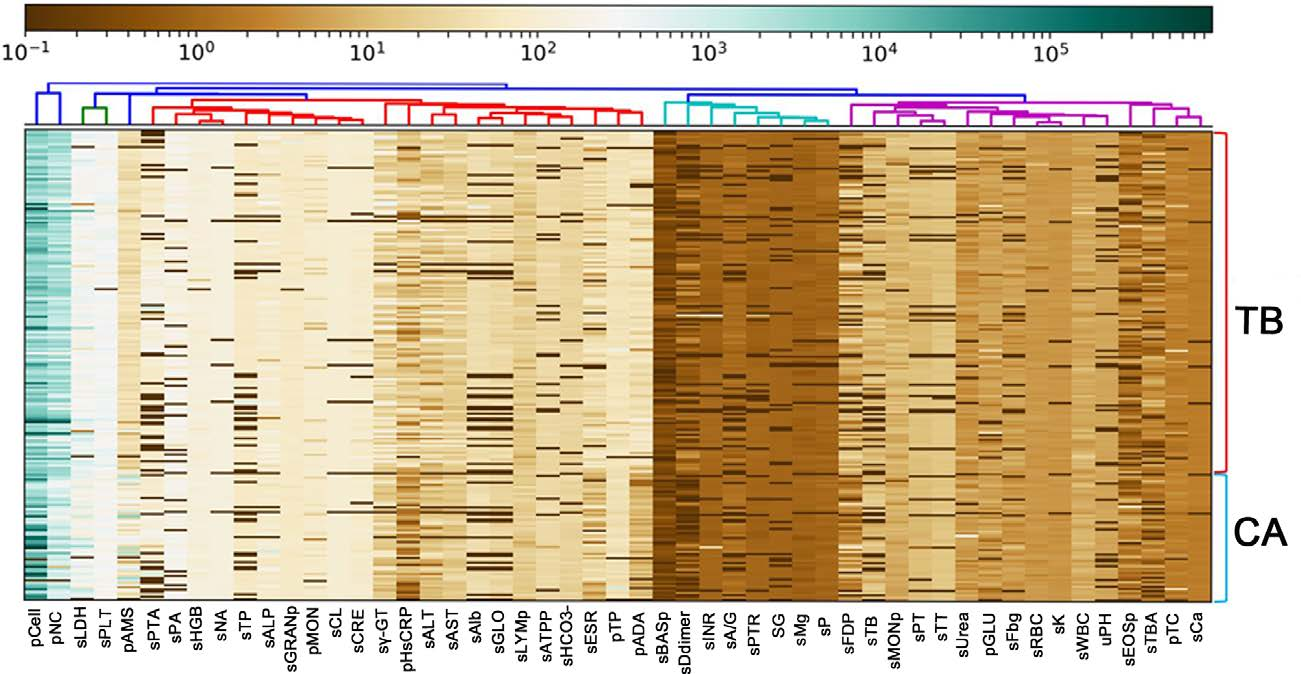
Patients with TB and CA were grouped by unsupervised hierarchical clustering of blood and pleural effusion clinical indicators. CA= tumor, TB = tuberculosis.

The indicators in the blood and PE samples were examined to observe the clinical features of TPE and MPE. Patients with TB and tumor were separated using unsupervised hierarchical clustering, with heatmap shown in Figure 1. The results showed that the serum and PE indicators of patients with tumor compared with those of patients with TB are quite different (Fig. 1).

**Figure 2.**
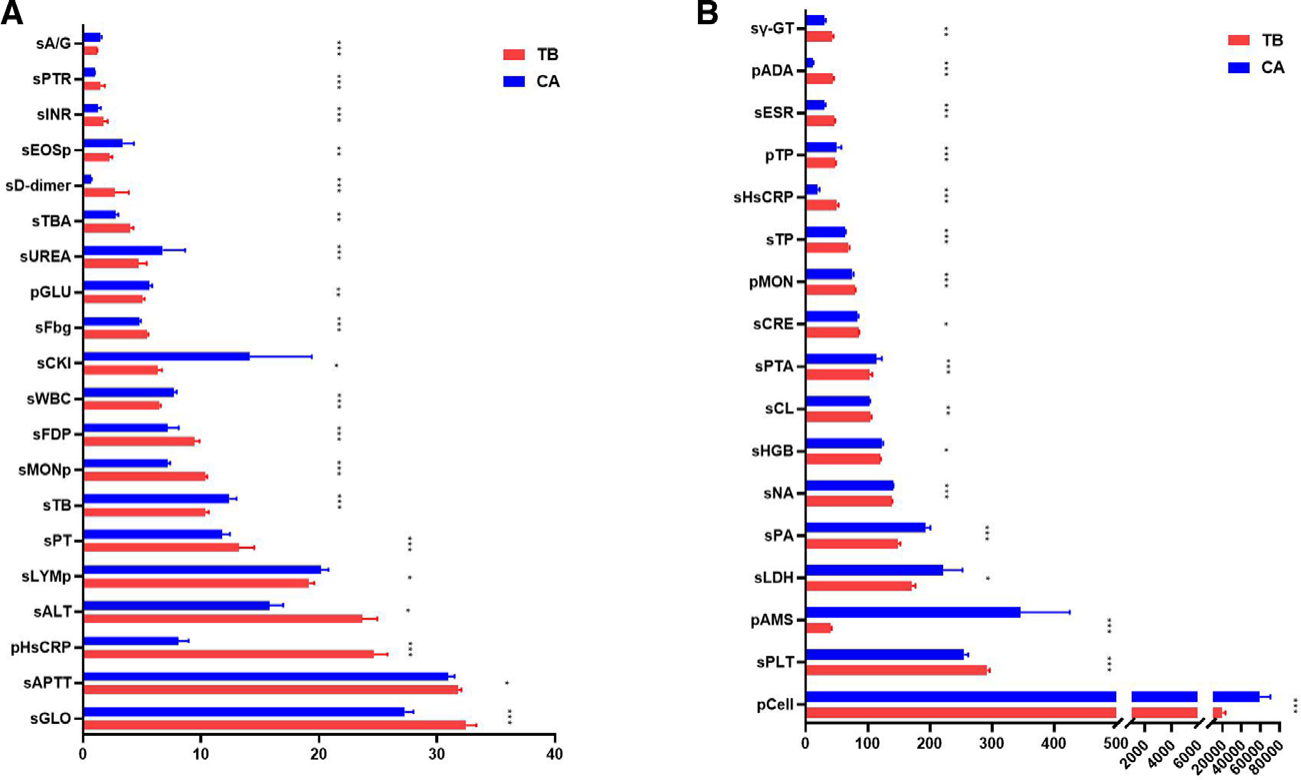
Blood and pleural effusion clinical indicators (N = 37) with statistical significance of TB and CA. (A) 20 indicators; (B) 17 indicators (mean value with SEM, *P < .05; **P < .01; ***P < .001). CA= tumor, TB = tuberculosis.

Blood parameters and PE indicators were compared and analyzed through Mann–Whitney *U* test to further obtain effective identification indicators (as shown in Table 3 and Fig. 2, 37 amongst 55 indicators between each group was statistically significant, *P *< .05). In serum, sESR, sMONp, sHsCRP and 16 other indicators were higher in the TPE group than in the MPE group (sESR: 46.27 ± 1.16 vs 29.78 ± 2.32, sMONp: 10.35 ± 0.17 vs 7.18 ± 0.20, sHsCRP: 50.30 ± 2.67 vs 19.25 ± 3.20; *P *< .01). Moreover, sWBC, sA/G, and 10 other indicators were higher in the MPE group than in the TPE group (sWBC: 7.70 ± 0.24 vs 6.48 ± 0.11, sA/G: 1.49 ± 0.08 vs 1.19 ± 0.02; *P *< .01). Amongst the indicators of PE, pADA, pHsCRP and pMON were higher in the TPE group than in the MPE group (pADA: 44.269 ± 0.997 vs 11.902 ± 0.969, pHsCRP: 24.63 ± 1.16 vs 8.07 ± 0.87, pMON: 79.66 ± 0.94 vs 75.33 ± 1.74; *P *< .01). In addition, hydrothorax amylase and 3 other markers were higher in the MPE group than in the TPE group (345.851 ± 79.170 vs 40.725 ± 1.023, *P *< .01; Fig. 2 and Table 3).

**Table 3:** Summary of indicators from comparative analysis of serum and pleural effusion.

Indicators	Diagnosis	N	Mean	SEM	Mann–Whitney *U* test (Sig.)
pCell	TB	567	19858.46	3463.52	<0.001
	CA	144	59114.03	11069.29	
pMON	TB	563	79.66	0.94	<0.001
	CA	147	75.33	1.74	
pTP	TB	547	47.99	0.79	<0.001
	CA	142	50.44	6.93	
pGLU	TB	548	5.05	0.16	0.001
	CA	142	5.59	0.26	
pAMS	TB	546	40.73	1.02	<0.001
	CA	142	345.85	79.17	
pHsCRP	TB	548	24.63	1.16	<0.001
	CA	142	8.07	0.87	
pADA	TB	547	44.27	1.00	<0.001
	CA	141	11.90	0.97	
sPTA	TB	450	102.62	4.66	<0.001
	CA	121	114.07	8.63	
sPT	TB	537	13.19	1.31	<0.001
	CA	151	11.78	0.66	
sPTR	TB	523	1.46	0.38	<0.001
	CA	146	0.99	0.01	
sINR	TB	537	1.72	0.36	<0.001
	CA	151	1.26	0.24	
sAPTT	TB	537	31.81	0.25	0.013
	CA	152	30.95	0.53	
sFbg	TB	537	5.44	0.08	<0.001
	CA	152	4.77	0.14	
sD-dimer	TB	490	2.69	1.19	<0.001
	CA	141	0.67	0.07	
sFDP	TB	490	9.45	0.42	<0.001
	CA	141	7.17	0.94	
sESR	TB	530	46.25	1.16	<0.001
	CA	136	29.78	2.32	
sWBC	TB	545	6.48	0.11	<0.001
	CA	155	7.70	0.24	
sLYMp	TB	545	19.14	0.45	0.037
	CA	155	20.16	0.64	
sEOSp	TB	545	2.24	0.22	0.003
	CA	155	3.37	0.94	
sMONp	TB	545	10.35	0.17	<0.001
	CA	155	7.18	0.20	
sHGB	TB	545	120.09	0.84	0.037
	CA	155	123.27	1.55	
sPLT	TB	545	292.18	4.25	<0.001
	CA	155	255.16	6.38	
sNA	TB	492	139.38	0.32	<0.001
	CA	127	140.74	0.37	
sCL	TB	491	104.42	1.85	0.001
	CA	127	103.39	0.40	
sALT	TB	512	23.65	1.28	0.029
	CA	123	15.82	1.14	
s*γ*-GT	TB	512	42.48	2.30	0.001
	CA	123	29.89	2.68	
sTP	TB	425	69.07	1.72	<0.001
	CA	102	64.01	0.67	
sGLO	TB	423	32.46	0.87	<0.001
	CA	102	27.26	0.73	
sA/G	TB	423	1.19	0.02	<0.001
	CA	102	1.49	0.08	
sTB	TB	426	10.37	0.28	<0.001
	CA	104	12.38	0.62	
sTBA	TB	454	4.00	0.27	0.001
	CA	106	2.74	0.24	
sPA	TB	437	148.70	3.70	<0.001
	CA	102	193.11	7.51	
sUREA	TB	495	4.69	0.70	<0.001
	CA	127	6.75	1.89	
sCRE	TB	494	85.45	0.75	0.028
	CA	127	83.70	1.56	
sCKI	TB	58	6.33	0.35	0.038
	CA	24	14.13	5.26	
sLDH	TB	61	170.62	6.02	0.024
	CA	24	221.54	31.01	
sHsCRP	TB	301	50.30	2.67	<0.001
	CA	70	19.25	3.20	

pADA = hydrothorax adenosine dehydrogenase, pAMS = hydrothorax amylase, pCell = hydrothorax cells, pGLU = hydrothorax glucose, pHsCRP = hydrothorax high sensitivity C reactive protein, pMON = hydrothorax monocytes, pTP = hydrothorax total protein, sA/G = blood A/G, sALT = alanine aminotransferase, sCa = serum calcium, sCK = blood creatine kinase, sCKI = blood creatine kinase isoenzyme, sCL = blood chlorine, sCRE = serum creatinine, sD-dimer = blood D-dimer, sEOSp = percentage of eosinophil, sESR = erythrocyte sedimentation rate, sFbg = blood fibrin, sFDP = fibrinogen degradation products, sGLO = globin, sHGB = hemoglobin, sHsCRP = blood high sensitivity C reactive protein, sINR = blood internationalization standardized ratio, sLDH = serum lactate dehydrogenase, sLYMp = lymphocyte percentage, sMONp = percentage of blood monocyte, sNA = blood natrium, sPA = blood prealbumin, sPLT = platelet, sPT = prothrombin time, sPTA = prothrombin activity, sPTR = prothrombin time ratio, sTB = total bilirubin, sTBA = total bile acid, sTP = blood total protein, sTT = blood thrombin time, sUREA = blood urea, sWBC = white blood cell, s*γ*-GT = blood *γ* glutamyl transpeptidase.

### 3.3. Effective markers to distinguish tuberculosis and malignant tumor with PE

**Figure 3.**
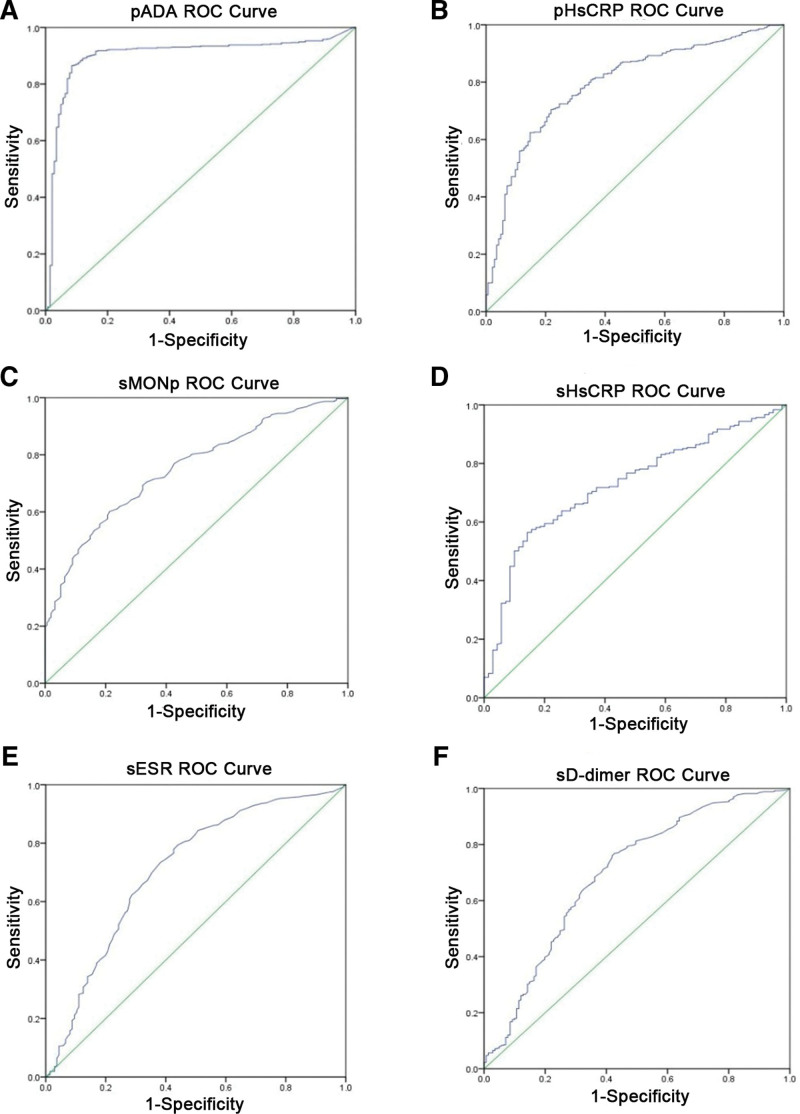
ROC analysis of blood and pleural effusion clinical indicators distinguishing TB from CA. (A) pADA ROC curve; (B) pHsCRP ROC curve; (C) sMONp ROC curve; (D) sHsCRP ROC curve; (E) sESR ROC curve; (F) sD-dimer ROC curve (AUC > 0.7). CA= tumor, pADA = hydrothorax adenosine dehydrogenase, pHsCRP = hydrothorax high sensitivity C reactive protein, sD-dimer = blood D-dimer, sESR = erythrocyte sedimentation rate, sHsCRP = blood high sensitivity C reactive protein, sMONp = percentage of blood monocyte, TB = tuberculosis.

**Figure 4.**
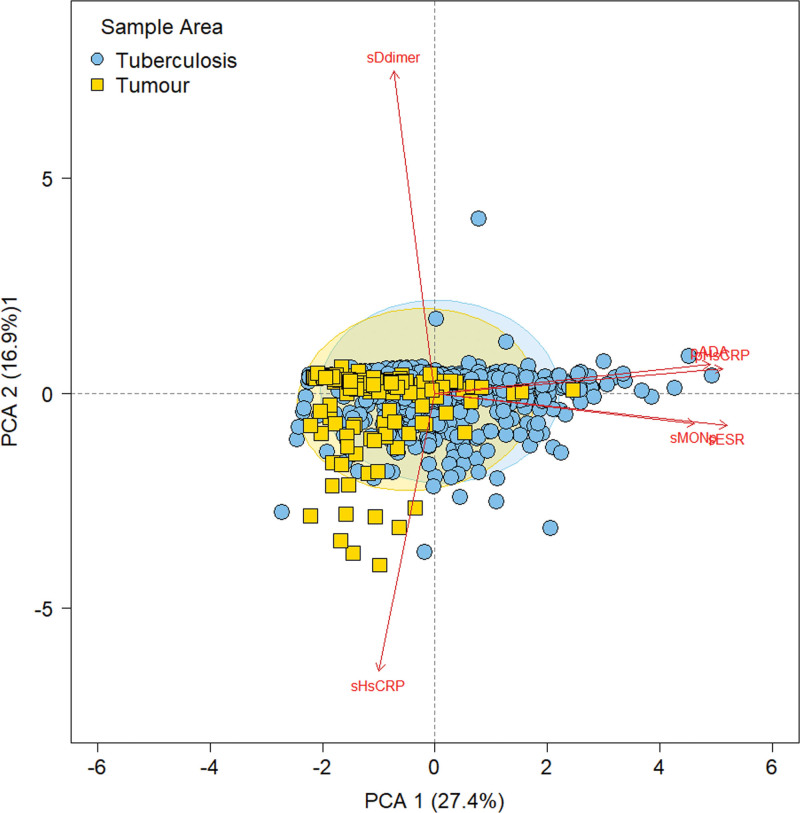
PCA analysis of 6 comparable blood and pleural effusion indicators of tuberculosis and tumor. PCA = principal component analysis.

The 37 indicators were applied to construct ROC curves to further screen effective diagnostic indicators. On the basis of the area AUC, sensitivity, and specificity), top 6 indicators with high diagnostic value were screened out (AUC ≥ 0.700; pADA, pHsCRP, sMONp, sHsCRP, sESR and sD-dimer; Fig. 3 and Table 4). In addition, principal component analysis (PCA) with these 6 serum or PE indicators revealed a clear separation between TB and malignant tumor (Fig. 4). Comparison between TB and tumor revealed that the pADA in PE showed the best AUC of 0.90 [95% confidence interval (CI): 0.87–0.93].

**Table 4:** ROC analysis of blood and pleural effusion indicators.

Features	AUC	Std. Error	95% Confidence Interval	Asymptotic Sig.
pADA	0.90	0.015	0.87–0.93	<0.001
pHsCRP	0.79	0.021	0.75–0.83	<0.001
sMONp	0.75	0.02	0.71–0.79	<0.001
sHsCRP	0.73	0.031	0.67–0.79	<0.001
sESR	0.71	0.027	0.66–0.76	<0.001
D-dimer	0.70	0.027	0.64–0.75	<0.001

D-dimer = blood D-dimer, pADA = hydrothorax adenosine dehydrogenase, pHsCRP = hydrothorax high sensitivity C reactive protein, sMONp = percentage of blood monocyte, sHsCRP = blood high sensitivity C reactive protein, sESR = erythrocyte sedimentation rate.

**Figure 5.**
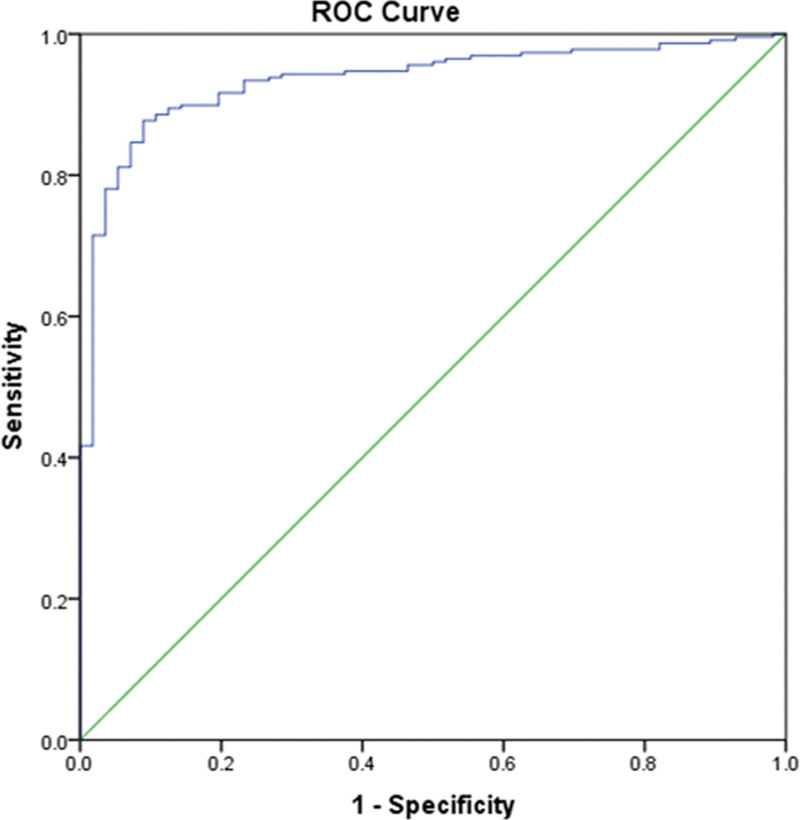
ROC analysis of combined indicators in tuberculosis detection form tumor patients.

The potential combination schemes of metabolic biomarkers based on logistic regression analysis were applied to enhance the sensitivity and accuracy of diagnosis of TB from malignant tumor with PE. As shown in Figure 5, the combination of 3 markers (pADA, sMONp and sHsCRP) remarkably enhanced the AUC to 0.944 (95% CI: 0.925–0.964). These results indicated that the 3 indicators could act as a promising combination for distinguishing TB from tumor with PE.

### 3.4. Diagnostic indicator of pADA for evaluation in different clinical patients’ characteristics

**Figure 6.**
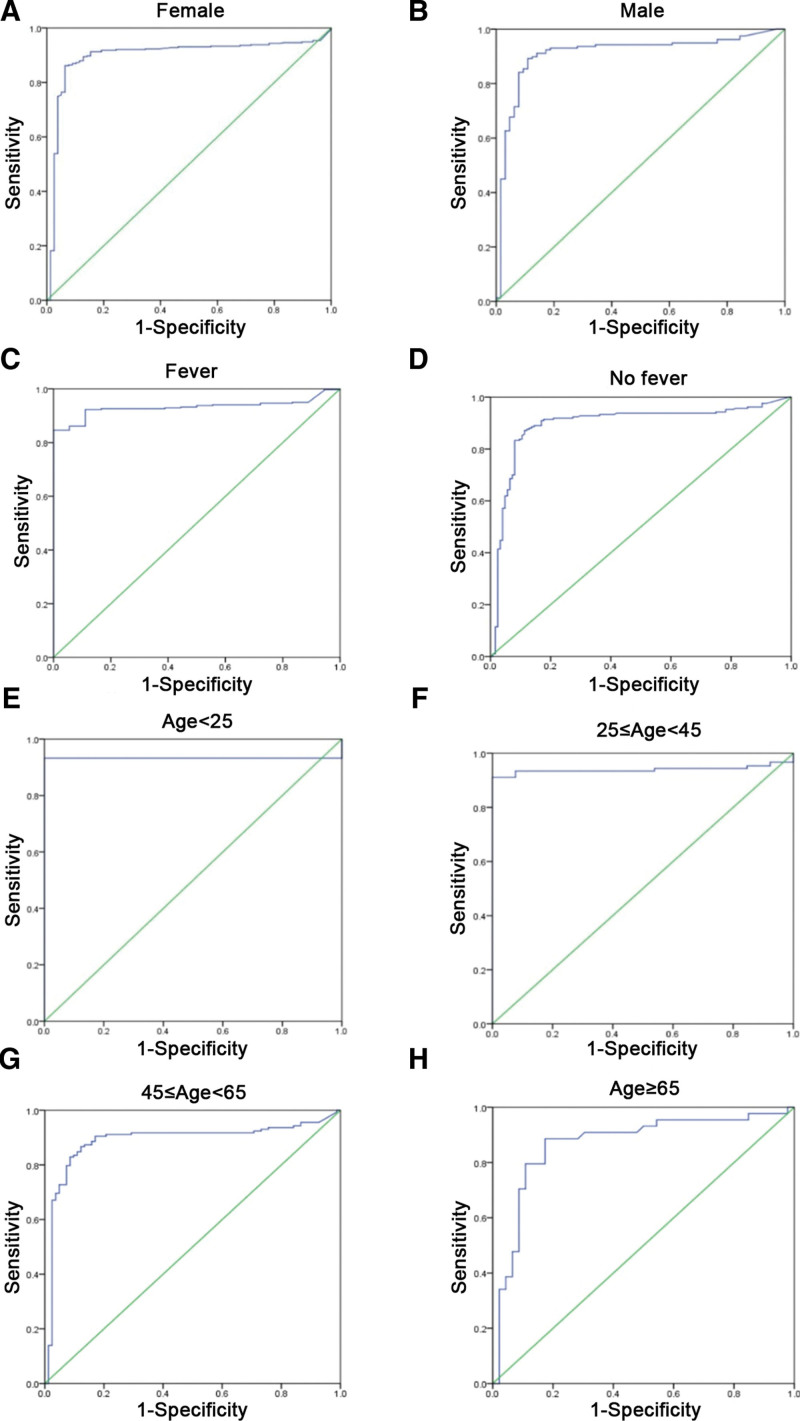
ROC analysis of selected indicator pADA in different statuses of patients. (A) pADA ROC curve (male); (B) pADA ROC curve (female); (C) pADA ROC curve (Fever); (D) pADA ROC curve (No fever); (E) pADA ROC curve (age < 25); (F) pADA ROC curve (25 ≤ age < 45); (G) pADA ROC curve (45 ≤ age < 65); (H) pADA ROC curve (age ≥ 65). pADA = hydrothorax adenosine dehydrogenase.

According to the above, the best indicator, namely, pADA, was close to the value of the combination of 3 markers (the only 1 ≥ 0.9). Thus, the alteration of indicators in different characteristics of patients was of great interest. In the stratified analysis, the AUC values of the pADA of males and females were 0.897 and 0.910. The AUC of patients with fever was 0.932, whilst that of patients without fever was 0.894, suggesting that the diagnosis accuracy of fever patients was higher. The AUC of age < 25 was 0.932, 25 ≤ age < 45 was 0.939, 45 ≤ age < 65 was 0.889 and age ≥ 65 was 0.868. Although with the increase in age, the AUC showed a downward trend and the diagnostic accuracy decreased (Fig. 6). The indicator pADA still showed the continuous distinguishing function as a diagnostic marker for TB and tumor with PE.

## 4. Discussion

In clinical practice, distinguishing TPE from MPE is very common and critical as the pathogenesis, treatment and recovery of the 2 diseases are different.^[18]^ Thus, early diagnosis is particularly important. In the present study, comparison between TPE and MPE showed that the patients of TPE were more likely to have fever and the MPE was bloodier (*P* < .01). Fever is commonly accepted by clinicians as a common symptom in patients with TPE.^[19]^ Other researchers proved that patients with TPE have a higher probability of fever than those with PE caused by malignant lymphoma (fever > 37.5°C, MPE = 12%, TPE = 48%, *P* < .01).^[20]^ Moreover, consistent with the present results, other clinicians observed that MPE is mostly bloody,^[21]^ which may be related to tumor invasion and the destruction of capillaries, leading to blood leakage.^[22]^

The percentage of positive Rivalta test for TPE was higher than that for MPE (*P* < .01). A study reported that the positive rate of Rivalta test is parallel to the amount of total protein in body cavity effusion.^[23]^ Some researchers showed that the protein level in TPE is higher than that in MPE (*P* < .05).^[24]^ Consistently, the present results showed that the blood total protein in TPE was higher than that in MPE (69.09 ± 1.72 vs 64.01 ± 0.67, *P *= .15), which could explain that the percentage of positive Rivalta test for TPE was higher than that for MPE.

In this study, serum D-dimer was higher in the TPE group than in the MPE group (2.69 ± 1.19 vs 0.67 ± 0.07, *P *= .37). Other researchers proved that the serum D-dimer level of patients with TPE was higher that of patients with MPE.^[14]^ However, they found that the difference of D-dimer in PE was more obvious.^[14]^ These findings showed that D-dimer is a highly sensitive index in serum and PE, thus helpful for identifying TPE and MPE.

sLDH was higher in the MPE group than in the TPE group (221.54 ± 31.01 vs 170.62 ± 6.02, *P *= .12). sLDH level was reported to be positively correlated with lymphoma-associated malignant PE (L-MPE, OR: 1.005, 95% CI: 1.003–1.007, *P *< .001). In addition, sLDH > 460 U/L distinguished L-MPE from TPE, with a sensitivity of 76% and a specificity of 81%.^[20]^ Consistent with the present results, other researchers observed that the sLDH in MPE was higher than that in TPE (*P* = .08).^[25]^ In multivariate logistic regression analysis, the ratio of sLDH to pleural fluid lymphocyte count was positively correlated with MPE. The sensitivity and specificity of this ratio were 0.63 (95% CI: 0.51–0.73) and 0.85 (95% CI: 0.68–0.94), respectively.^[25]^ Therefore, sLDH is an important indicator for distinguishing TPE from MPE.

High sensitivity C reactive protein (HsCRP) is widely used as a sensitive but nonspecific marker of systemic inflammation.^[26,27]^ Increased sHsCRP levels have been reported in many lung diseases, including tumors and TB.^[28,29]^ In the present study, the median levels of pHsCRP and sHsCRP were higher in the TPE group than in the MPE group (24.63 ± 1.16 vs 8.07 ± 0.87 and 50.30 ± 2.67 vs 19.25 ± 3.20, *P *< .01). The AUC values of pHsCRP and sHsCRP were 0.79 and 0.73, respectively. Consequently, HsCRP is an important reference indicator to differentiate TPE from MPE. A meta-analysis showed that the optimal critical value of pHsCPR was 21.9 mg/dL; the values above the critical value were classified as TPE and those below the critical value were classified as MPE, the sensitivity was 0.91 (0.73–0.98) and the specificity was 0.82 (0.7–0.9).^[30]^

Although HsCRP is a valuable diagnostic indicator, the diagnosis efficiency is low. Thus, the choice of multi-index joint analysis is conducive to improving the diagnosis efficiency and accuracy. Through logical analysis, 6 relevant indicators (pADA, pHsCRP, sD-dimer, sESR, sHsCRP and sMONp) were selected. The logistic regression model showed that 3 variables of pADA, sMONp and sHsCRP could better help distinguish patients with PE by TB from malignant tumor. The combined AUC of the 3 factors could reach 0.94 (95% CI: 0.91–0.97), higher than that of any single index. Therefore, they have great significance for the clinical differentiation between TPE and MPE. In agreement with the present study, 1 study analyzed 118 patients, including 84 patients with MPE (71.2%) and 34 patients with TPE (28.8%). The results showed that the pADA of TPE was higher than that of MPE (*P* < .05).^[25]^ Moreover, others have proven that elevated levels of sHsCRP and pADA in PE were useful in distinguishing TPE from MPE.^[31,32]^ However, only 1 study had a different result. After analyzing 17 patients with L-MPE and 216 patients with TPE, the authors found no statistically significant difference in sHsCRP and pADA levels between the 2 groups,^[20]^ and the reason could be related to the number of patients with MPE included in this study.

At present, to achieve enhanced treatment efficacy in clinical practice, many researchers were interested in exploring the differentiation between TPE and MPE.^[33–36]^ Some of them focused on inflammatory factors. The biomarkers of PE in 22 patients with MPE and 5 patients with TPE were compared. IL-1, IP-10, IL-13, and IFN-*γ* were significantly higher in TPE (*P* < .05). The level of basic fibroblast growth factor in MPE was higher than that in TPE (*P *< .05).^[33]^ The highest AUC was found in IP-10 (AUC = 0.95, 95% CI, *P *< .01), followed by IL-13 (AUC = 0.86, 95% CI, *P *< .05).^[33]^ However, though 1 of the indicators in this study showed a high AUC, detection is not a common clinical indicator, and it is complicated. The sample size was also small, the reliability was weak, and performing stratified analysis was difficult. Another study found that the fibronectin and cathepsin G in patients with MPE were significantly higher than those in patients with TPE, whilst leukotriene-a4 hydrolase was lower than in patients with TPE.^[34]^ The AUC was determined to be 0.285 for fibronectin (95% CI: 0.174–0.396), 0.64 for leukotriene-a4 hydrolase (95% CI: 0.518–0.762), 0.337 for cathepsin G (95% CI: 0.218–0.456), and 0.793 for a combination of these candidate markers (95% CI: 0.697–0.888). The AUC was significantly lower than that in the present study.

In this study, the results exhibited more significant advantages of high diagnostic accuracy (high AUC, high sensitivity and specificity) and large sample size, which indicate high data reliability. In addition, pADA, sHsCRP and sMONp are all clinically common and easy-to-collect specimens, which are convenient and cheap to test. Thus, they do not pose additional burden on patients. Hierarchical analysis could be performed because of the large sample size, and the results showed that the diagnostic efficiency of pADA differed in various age groups. As age increased, the diagnostic efficiency of pADA gradually decreased. This phenomenon could be related to the percentage of TB decreasing, whilst CA diagnosis increased with age. This finding suggested that patients under 45 years could choose the single indicator pADA for diagnostic detection. Age factors also affect changes in sMONp, and aging leads to a decrease in immune function, resulting in a decrease in the number and quality of monocytes in the blood.^[37]^ Meanwhile, factors such as age also affect changes in sHsCRP, with significant differences between older and younger people.^[38]^ These indicate that the diagnostic model constructed by the 3 indicators pADA, sHsCRP and sMONp, although having high diagnostic efficacy, can be influenced by various factors, and we will continue to validate and optimize the diagnostic indicators and their influencing factors in the subsequent studies.

The gold standard for differentiating TPE and MPE in clinical practice still relies on pathological tissue biopsy.^[39]^ All cases in the present study were examined by thoracoscopy, and pathological biopsy was completed in most cases, ensuring the accuracy of diagnosis. However, for some patients who refuse to accept the invasive examination or whose constitution is difficult to bear invasive examination, the effective detection index of noninvasive examination provides a strong basis for timely diagnosis and accurate treatment. Further research and exploration are worth conducting.

## 5. Conclusion

In summary, the results showed some noninvasive and valuable markers for differentiating TPE from MPE. Although the gold standard for differentiating TPE and MPE still relies on pathological tissue biopsy, for some patients who refuse to accept invasive examination or whose constitution is difficult to bear invasive examination, the effective detection index of noninvasive examination provides a strong basis for timely diagnosis and accurate treatment.

## Authors’ contributions

Conception and design: ELHL, RZL, MFW, XJY; Administrative support: LL, YJT; Provision of study materials or patients: MFW, TR, ZXF, XXF, HDP; Collection and assembly of data: MFW; Data analysis and interpretation: RZL, WYM, JW, IK; Manuscript writing: All authors; Final approval of manuscript: All authors.

**Conceptualization:** Liang Liu, Xiao-Jun Yao, Run-Ze Li, Mei-Fang Wang, Elaine Lai-Han Leung.

**Data curation:** Jian Wang, Zhe-Xiang Feng, Tao Ren, Wei-Yu Meng, Imran Khan, Yi-Jun Tang, Mei-Fang Wang.

**Formal analysis:** Jian Wang, Wei-Yu Meng, Imran Khan, Yi-Jun Tang, Run-Ze Li.

**Writing – original draft:** Jian Wang.

**Writing – review & editing:** Jian Wang, Zhe-Xiang Feng, Tao Ren, Xing-Xing Fan, Hu-Dan Pan, Liang Liu, Xiao-Jun Yao, Run-Ze Li, Mei-Fang Wang, Elaine Lai-Han Leung.

## References

[1] Zhang F Wang J Zheng X. Clinical value of jointly detection pleural fluid Midkine, pleural fluid adenosine deaminase, and pleural fluid carbohydrate antigen 125 in the identification of nonsmall cell lung cancer‐associated malignant pleural effusion. J Clin Lab Anal. 2018;32:e22576. 29797475 10.1002/jcla.22576 PMC6817252

[2] Garske LA Kunarajah K Zimmerman PV. In patients with unilateral pleural effusion, restricted lung inflation is the principal predictor of increased dyspnoea. PLoS One. 2018;13:e0202621. 30281613 10.1371/journal.pone.0202621 PMC6169850

[3] Doelken P. Clinical implications of unexpandable lung due to pleural disease. Am J Med Sci. 2008;335:21–5. 18195579 10.1097/MAJ.0b013e31815f1a44

[4] Sahn S Huggins J San José M. Can tuberculous pleural effusions be diagnosed by pleural fluid analysis alone? Int J Tuberc Lung Dis. 2013;17:787–93. 23676163 10.5588/ijtld.12.0892

[5] Lange C Mori T. Advances in the diagnosis of tuberculosis. Respirology. 2010;15:220–40. 20199641 10.1111/j.1440-1843.2009.01692.x

[6] Porcel JM Gasol A Bielsa S. Clinical features and survival of lung cancer patients with pleural effusions. Respirology. 2015;20:654–9. 25706291 10.1111/resp.12496

[7] Mcgrath EE Warriner D Anderson PB. Pleural fluid characteristics of tuberculous pleural effusions. Heart Lung. 2010;39:540–3. 20561884 10.1016/j.hrtlng.2009.12.004

[8] Maskell N Butland R, Pleural Diseases Group, Standards of Care Committee, British Thoracic Society. BTS guidelines for the investigation of a unilateral pleural effusion in adults. Thorax. 2003;58(Suppl. 2):ii8–ii17. 12728146 10.1136/thorax.58.suppl_2.ii8 PMC1766019

[9] Gao B-A Zhou G Guan L. Effectiveness and safety of diagnostic flexi-rigid thoracoscopy in differentiating exudative pleural effusion of unknown etiology: a retrospective study of 215 patients. J Thor Dis. 2014;6:438–43. 10.3978/j.issn.2072-1439.2014.02.09 PMC4015023 24822100

[10] Zhang M Li D Hu Z-D. The diagnostic utility of pleural markers for tuberculosis pleural effusion. Ann Transl Med. 2019;8:607. 10.21037/atm.2019.09.110 PMC7290547 32566633

[11] Helmy NA Eissa SA Masoud HH. Diagnostic value of adenosine deaminase in tuberculous and malignant pleural effusion. Egypt J Chest Dis Tuberculosis. 2012;61:413–7.

[12] Goto M Noguchi Y Koyama H. Diagnostic value of adenosine deaminase in tuberculous pleural effusion: a meta-analysis. Ann Clin Biochem. 2003;40:374–81. 12880538 10.1258/000456303766477011

[13] Samanta S Sharma A Das B. Significance of total protein, albumin, globulin, serum effusion albumin gradient and LDH in the differential diagnosis of pleural effusion secondary to tuberculosis and cancer. J Clin Diagn Res. 2016;10:BC14. 10.7860/JCDR/2016/20652.8379 PMC5028544 27656432

[14] Shen Y Yang T Jia L. A potential role for D-dimer in the diagnosis of tuberculous pleural effusion. Age. 2013;17:201–5. 23377808

[15] Antonangelo L Vargas FS Seiscento M. Clinical and laboratory parameters in the differential diagnosis of pleural effusion secondary to tuberculosis or cancer. Clinics. 2007;62:585–90. 17952319 10.1590/s1807-59322007000500009

[16] Chen M-L Yu W-C Lam C-W. Diagnostic value of pleural fluid adenosine deaminase activity in tuberculous pleurisy. Clin Chim Acta. 2004;341:101–7. 14967164 10.1016/j.cccn.2003.11.016

[17] Radjenovic-Petkovic T Pejcic T Nastasijevic-Borovac D. Diagnostic value of CEA in pleural fluid for differential diagnosis of benign and malign pleural effusion. Med Arh. 2009;63:141–2. 20088159

[18] Jin D Chen Y Wang Z. Diagnostic value of interleukin 22 and carcinoembryonic antigen in tuberculous and malignant pleural effusions. Exp Ther Med. 2011;2:1205–9. 22977645 10.3892/etm.2011.344 PMC3440797

[19] Zhai K Lu Y Shi H-Z. Tuberculous pleural effusion. J Thor Dis. 2016;8:E486–94. 10.21037/jtd.2016.05.87 PMC4958858 27499981

[20] Kim CH Oh HG Lee SY. Differential diagnosis between lymphoma-associated malignant pleural effusion and tuberculous pleural effusion. Ann Transl Med. 2019;7:373. 31555687 10.21037/atm.2019.07.17 PMC6736794

[21] Karkhanis VS Joshi JM. Pleural effusion: diagnosis, treatment, and management. Open Access Emerg Med. 2012;4:31–52. 27147861 10.2147/OAEM.S29942 PMC4753987

[22] Chikileva IO Anisimova NY Shubina IZ. Pathogenesis of Malignant Effusions. Malignant Effusions. Springer. 2012: 11–21.

[23] Zhu S Du L Xu D. Ascitic fluid total protein, a useful marker in non-portal hypertensive ascites. J Gastroenterol Hepatol. 2020;35:271–7. 31247673 10.1111/jgh.14768

[24] Umar M Iqbal Z Basit A. Validity of pleural fluid protein in differentiating tuberculous from malignant pleural effusion. Pak J Chest Med. 2018;24:141–6.

[25] Verma A Dagaonkar RS Marshall D. Differentiating malignant from tubercular pleural effusion by cancer ratio plus (cancer ratio: pleural lymphocyte count). Can Respir J. 2016;2016:7348239. 28070157 10.1155/2016/7348239 PMC5192296

[26] Park D-S Kim D Hwang K-E. Diagnostic value and prognostic significance of pleural C-reactive protein in lung cancer patients with malignant pleural effusions. Yonsei Med J. 2013;54:396–402. 23364973 10.3349/ymj.2013.54.2.396 PMC3575996

[27] Pepys MB Hirschfield GM. C-reactive protein: a critical update. J Clin Invest. 2003;111:1805–12. 12813013 10.1172/JCI18921 PMC161431

[28] Allin KH Nordestgaard BG. Elevated C-reactive protein in the diagnosis, prognosis, and cause of cancer. Crit Rev Clin Lab Sci. 2011;48:155–70. 22035340 10.3109/10408363.2011.599831

[29] Yoon C Chaisson LH Patel SM. Diagnostic accuracy of C-reactive protein for active pulmonary tuberculosis: a meta-analysis. Int J Tuberc Lung Dis. 2017;21:1013–9. 28826451 10.5588/ijtld.17.0078 PMC5633000

[30] Angeles RRB King RE Benedicto J. Accuracy of pleural fluid c-reactive protein in differentiating between tuberculous and malignant pleural effusions: a meta-analysis. Eur Resp Soc. 2019:PA2978.

[31] Lee J Lee YD Lim JK. Predictive factors and treatment outcomes of tuberculous pleural effusion in patients with cancer and pleural effusion. Am J Med Sci. 2017;354:125–30. 28864369 10.1016/j.amjms.2017.04.006

[32] Petborom P Dechates B Muangnoi P. Differentiating tuberculous pleuritis from other exudative lymphocytic pleural effusions. Ann Palliat Med. 2020;9:2508–15. 32921071 10.21037/apm-19-394

[33] Chen K-Y Feng P-H Chang C-C. Novel biomarker analysis of pleural effusion enhances differentiation of tuberculous from malignant pleural effusion. Int J Gen Med. 2016;9:183–9. 27354819 10.2147/IJGM.S100237 PMC4910680

[34] Shi J Li P Zhou L. Potential biomarkers for antidiastole of tuberculous and malignant pleural effusion by proteome analysis. Biomarkers Med. 2019;13:123–33. 10.2217/bmm-2018-0200 30791695

[35] Liu Y Mei B Chen D Cai L. GC-MS metabolomics identifies novel biomarkers to distinguish tuberculosis pleural effusion from malignant pleural effusion. J Clin Lab Anal. 2021;35:e23706. 33528039 10.1002/jcla.23706 PMC8059743

[36] Robak A Kistowski M Wojtas G. Diagnosing pleural effusions using mass spectrometry-based multiplexed targeted proteomics quantitating mid- to high-abundance markers of cancer, infection/inflammation and tuberculosis. Sci Rep. 2022;12:3054. 35197508 10.1038/s41598-022-06924-y PMC8866415

[37] De Maeyer R PH Chambers ES. The impact of ageing on monocytes and macrophages. Immunol Lett. 2021;230:1–10. 33309673 10.1016/j.imlet.2020.12.003

[38] Marcos-Pérez D Sánchez-Flores M Proietti S. Association of inflammatory mediators with frailty status in older adults: results from a systematic review and meta-analysis. GeroScience. 2020;42:1451–73. 32803650 10.1007/s11357-020-00247-4 PMC7732924

[39] Liu Q Yu Y-X Wang X-J. Diagnostic accuracy of interleukin-27 between tuberculous pleural effusion and malignant pleural effusion: a meta-analysis. Respiration. 2018;95:469–77. 29539604 10.1159/000486963

